# Increased Dipeptidyl Peptidase‐4 Promotes Adipose Inflammation and Dysfunction in Mice Under Chronic Stress

**DOI:** 10.1096/fj.202502147R

**Published:** 2025-07-31

**Authors:** Meiping Zhang, Huazhen Wang, Xiangdan Li, Shangzhi Shu, Jinshun Piao, Xueling Yue, Songzhen Zhao, Miao Li, Xianglan Jin, Yongshan Nan, Xian Wu Cheng

**Affiliations:** ^1^ Department of Cardiology and Hypertension Jilin Provincial Key Laboratory of Stress and Cardiovascular Disease, Yanbian University Hospital Yanji Jilin P.R. China; ^2^ Department of Anesthesiology Yanbian University Hospital Yanji Jilin P.R. China; ^3^ Department of Morphological Experiment Center Medical College of Yanbian University Yanji Jilin P.R. China; ^4^ Department of Cardiovascular Disease The First Hospital of Jilin University Changchun Jilin P.R. China; ^5^ Key Laboratory of Cellular Function and Pharmacology of Jilin Province Yanbian University Yanji Jilin P.R. China

**Keywords:** apoptosis, chronic stress, dipeptidyl peptidase‐4, glucagon‐like peptide‐1, inflammation

## Abstract

Exposure to chronic psychological stress is an intractable risk factor for inflammatory and metabolic disorders. Given that dipeptidyl peptidase‐4 (DPP4) is upregulated in stressed adipose and vascular tissues and modulates intracellular signaling pathways related to glucagon‐like peptide‐1 (GLP‐1) metabolism, we investigated the role of the DPP4/GLP‐1 axis by examining adipose inflammation and dysfunction in mice subjected to chronic stress. Eight‐week‐old male wild‐type mice (DPP4^+/+^) and DPP4‐knockout (DPP4^−/−^) mice were randomly assigned to non‐stress or 2‐week immobilization stress groups for morphological and biochemical analyses. On Day 14 post‐stress, the stressed mice had reduced subcutaneous adipose tissue (SWAT) weights and detrimental changes in the protein and/or mRNA levels of phospho‐phosphatidylinositol 3‐kinase, phospho‐protein kinase B, oxidative stress (gp91^phox^)‐, apoptosis (cytochrome *c*, caspase‐8, B‐cell lymphoma 2 and Bcl2‐associated X protein)‐, monocyte chemoattractant protein‐1, interleukin‐6, tumor‐necrosis factor‐α, and inducible nitric oxide synthase‐related molecules, plus increased F4/80^+^ macrophage infiltration; these changes were reversed by DPP4 deficiency. The GLP‐1 receptor agonist exenatide mimicked the adipose benefits of DPP4 deletion. In vitro, exenatide reduced the production of reactive oxygen species and the apoptosis induced by stressed serum, and it altered the levels of p‐AKT protein. DPP4 deletion also reduced these changes. These findings suggest that DPP4 functions as an important mediator of chronic stress‐stimulated adipose inflammation and dysfunction via the modulation of adipocyte oxidative stress production and apoptosis that is mediated by the GLP‐1/PI3K‐AKT axis, indicating that DPP4 inhibition and/or GLP‐1R stimulation may have applications for treating metabolic disorders of patients under chronic stress conditions.

## Graphical abstract

Graphical illustration of mechanism in adipose inflammation. DPP4 functions as an important mediator of chronic stress‐stimulated adipose inflammation and dysfunction via the modulation of adipocyte oxidative stress production and apoptosis that is mediated by the GLP‐1/PI3K‐AKT axis.

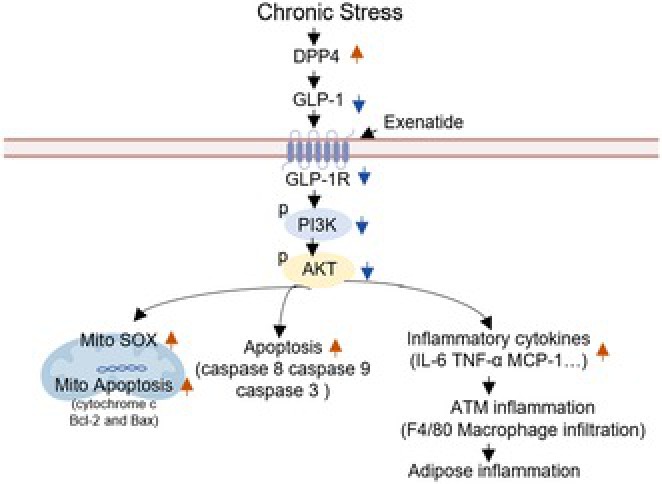

**Abbreviations**
AKT: protein kinase B
ATM: adipose tissue macrophage
BW: body weight
CLS: crown‐like structure
CPS: chronic psychological stress
CXCR4: CXC chemokine receptor 4
DMEM: Dulbecco's modified eagle medium
DPP4: dipeptidyl peptidase‐4
GAPDH: glyceraldehyde 3‐phosphate dehydrogenase
GLP‐1: glucagon‐like peptide‐1
H&E: hematoxylin and eosin
IL‐6: interleukin‐6
iNOS: inducible nitric oxide synthase
MCP‐1: monocyte chemotactic protein 1
p‐AKT^ser473^
: phosphorylated‐Akt‐(Ser473)
PI3K: phosphoinositide 3‐kinase
p‐PI3K: phospho‐PI3K p85 (Tyr458)/p55
PVDF: polyvinylidene difluoride
qPCR: quantitative polymerase chain reaction
RNA‐Seq: RNA sequencing
ROS: reactive oxygen species
SDF‐1: stromal cell‐derived factor‐1
SWAT: subcutaneous white adipose tissue
TNF‐α: tumor necrosis factor‐alpha

## 1 Introduction

Over the past two decades, mounting evidence has highlighted chronic psychological stress (CPS) as a significant contributor to the rising prevalence of inflammatory and metabolic disorders including diabetes, atherosclerosis, and cancer [1, 2, 3, 4]. Notably, recent studies have demonstrated that CPS results in adipocyte dedifferentiation and adipogenesis, leading to adipose remodeling and dysfunction [5, 6]. However, the underlying molecular mechanisms of these findings are largely uncertain.

Adipose tissue macrophages (ATMs) are major targets of adipose inflammation and are distributed in large numbers [7, 8]. In obesity, ATMs are recruited from blood circulation to form distinctive crown‐like structures (CLSs) around dying or dead adipocytes; the ATMs are closely associated with chronic inflammation, which disrupts glucose, lipid, and energy metabolism [8, 9]. Adipose tissue in individuals with obesity is heavily infiltrated by classically activated macrophages (M1) and T‐cell subpopulations, both of which secrete pro‐inflammatory cytokines such as monocyte chemoattractant protein‐1 (MCP‐1), tumor‐necrosis factor‐alpha (TNF‐α), and interleukin (IL)‐6 [10, 11, 12]. It was reported that CPS can perturb the equilibrium of the immune system, amplify inflammation, and attenuate immune surveillance, thereby facilitating the emergence of a spectrum of metabolic diseases (e.g., obesity and diabetes) [13, 14]. It is thus very important to understand the exact mechanisms by which CPS interferes with adipose inflammation and dysfunction.

Dipeptidyl peptidase‐4 (DPP4, also known as T cell‐activating antigen CD26) is originally widely expressed in adipose tissues and their stromal/stem cells [15]. DPP4 has been shown to be significantly upregulated in various proinflammatory conditions including obesity, diabetes, thrombosis, and atherosclerotic vascular diseases [16, 17, 18, 19, 20]. This upregulation is closely associated with the underlying inflammatory and apoptosis processes and contributes to the pathophysiology of these conditions [21, 22]. DPP4 activity regulates inflammation by the proteolytic cleavage of immunoregulatory peptides and via its soluble form that directly drives immune cells [22]. DPP4 deficiency has been shown to significantly attenuate the ability to activate macrophages and influence their capacity to secrete proinflammatory cytokines [23, 24, 25]. It was also reporte4d that functionally mature macrophages had elevated expressions of CD68 and DPP4 via the nuclear factor‐κB mechanism [26, 27]. One of our research group's recent investigations demonstrated that stress leads to vascular senescence, inflammation, and atherosclerotic plaque formation through a negative regulation of the activation of signaling by both GLP‐1 and its receptor that is mediated by DPP4 [16, 17, 18, 19, 20, 28]. However, the exact mechanisms that underlie chronic stress‐related adipose inflammation and dysfunction remain largely unknown.

We conducted the present study to examine the pathogenic role of DPP4, using wild‐type (WT) and DPP4‐knockout (DPP4^−/−^) mice as chronic stress models together with a non‐stress condition (control mice), followed by morphological and biological analyses. In a separate experiment, WT mice subjected to CPS received a vehicle or a specific GLP‐1 receptor agonist, i.e., exenatide (S‐Exe). To further investigate the molecular mechanisms, we used a stressed serum (S‐serum)‐induced cellular model and examined the intracellular signaling pathways by omitting and adding exenatide as well as a PI3K/AKT inhibitor, subjected to biological analyses.

## 2 Materials and Methods

### 2.1 Network Pharmacology Analysis

The chemical structure of exenatide was imported into the Swiss Target Prediction and Super Pred databases for a search for potential exenatide targets. After the removal and merging of duplicates, the results were considered potential targets of exenatide. We also used the keyword phrase “chronic stress or adipose inflammation” to look for disease‐related targets in the Gene Cards and DisGeNET databases. The retrieved molecules were merged, duplicates were removed, and targets related to adipose inflammation were further identified based on a literature review [29].

A protein–protein interaction (PPI) network was constructed and analyzed [29]. The intersection of exenatide component targets and chronic stress‐ or adipose‐inflammation‐related targets was identified. These intersecting targets were imported into the STRING database with the species set to *Homo sapiens* and the minimum required interaction score set to medium confidence (0.400). The resulting TSV file was imported into Cytoscape 3.10.2 for visualization and evaluation. We applied the Network Analyzer plugin to examine topological parameters and targets with a degree value equal to or higher than the mean, and these parameters and targets were refined as core molecular targets for exenatide in the management of chronic stress and adipose inflammation.

A KEGG pathway enrichment analysis was performed as described [30]. In brief, key molecule targets obtained from the intersection of drug and disease targets were imported into the Metascape database for the KEGG pathway enrichment analysis. Visualization was achieved by using the Omicshare platform (https://www.omicshare.com/), with the species restricted to a significance threshold of *p* < 0.05 and the species set as *Homo sapiens* . The abundant genes were graded according to their enrichment scores in order to obtain the biological information and functional annotations of the key molecule targets, and we then analyzed the potential mechanisms of the actions of exenatide in the treatment of chronic stress and adipose inflammation.

### 2.2 Data Mining

We conducted data mining of the RNA sequencing original data from GSE228094 in the GEO database, which contains the RNA sequencing data of seven groups: adipocyte mono (A_mono), adipocyte with M1 macrophages (A_with_M1), adipocyte with M2 macrophages (A_with_M2), M1 mono, M2 mono, M1 with adipocytes (M1_with_A), and M2 with adipocytes (M2_with_A).

### 2.3 Ethical Approval and Animal Housing

All animal procedures were performed in strict accordance with the Animal Research Ethics Committee of Yanbian University Medical College (protocol no. YD20240430002) and the U.S. NIH guidelines for the care and use of laboratory animals [31]. Male DPP4^+/+^ (C57BL/6J, i.e., WT) mice and DPP4^−/−18^ mice aged 8 weeks and weighing 22–26 g were used in the experiments. The mice were housed in a controlled environment (22°C ± 2°C, 50% ± 5% humidity) and fed a standard diet and distilled water under a 12‐h light/dark cycle. The mice were continuously monitored throughout the study by trained research staff.

### 2.4 Chronic Stress Procedures

The DPP4^+/+^ and DPP4^−/−^ mice were randomly assigned to non‐stress groups (NS‐ DPP4^+/+^ and NS‐DPP4^−/−^) and stress groups (S‐DPP4^+/+^ and S‐DPP4^−/−^). Non‐stress groups of mice of each strain were left undisturbed and placed in separately ventilated cages. Each of the stress groups was subjected to variable restraint stress for 4 h (10 a.m. to 2 p.m.) daily in an immobilization stress cage (cat. no. G‐13302, Natsume Seisakusho, Tokyo) for 2 consecutive weeks. The restraint stress protocol was performed as described [6].

We applied four different stress paradigms over the 2‐week period: cage tilt, isolation, horizontal caging and damping, and overnight lighting. The order of these stressors was randomized. For the cage tilt stress, the mouse was placed in a cage tilted at a 45° angle for 4 h. For isolation, the mouse was individually housed in the immobilization stress cage mentioned above. For horizontal caging and damping, sawdust was removed from the cage, water was added, and the cage was suspended horizontally with the mouse's tail in the water for 4 h. For overnight lighting, the mouse was exposed to continuous light from 9 p.m. to 9 a.m. in a room that was separate from the home cages.

In separate GLP‐1 receptor agonist experiments, DPP4^+/+^ mice were assigned to three groups and given (by oral gavage) either a vehicle (saline, control, C‐NaCl and saline, stress, or S‐NaCl) or a dose of the GLP‐1R agonist exenatide (S‐Exe, 5 μg/kg/d) daily, with continued daily restraint stress for 2 weeks.

### 2.5 Sample Collection and Processing

**FIGURE 1:**
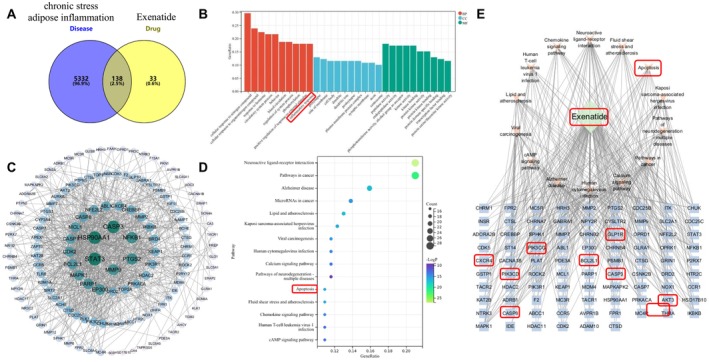
A network pharmacology analysis was performed to identify target molecules of the exenatide‐mediated prevention of chronic stress‐related adipose inflammation. (A) A Venn diagram of exenatide and chronic stress‐related adipose inflammation, which includes 171 exenatide‐related targets (right), 5470 chronic stress‐related adipose inflammation targets (left), and 138 overlapping targets (center). (B) The gene ontology (GO) pathway enrichment analysis. (C) The anti‐adipose inflammation protein–protein interaction (PPI) network. A larger area indicates larger nodes, blue indicates a closer association, lighter color indicates a less close association, and the core target is the target in the inner circle. (D) The KEGG pathway enrichment analysis (DAVID), with the pathways (*y*‐axis), false discovery rate (FDR) (*x*‐axis), and *p*‐values (color change). The bubble size indicates the number of genes enriched in the pathway. (E) The active ingredients‐key targets‐pathway PPI network.

On the night before sample collection, the food and water were removed from the home cages. At the indicated time points, mice were euthanized with 5% isoflurane at the Animal Center of Yanbian University Graduate School of Medicine [32]. Blood samples and subcutaneous adipose tissues were then collected (Figure 1A). For the morphological analysis, subcutaneous white adipose tissue (SWAT) was immediately embedded in paraffin and processed for hematoxylin and eosin (H&E) staining (Solarbio, Beijing, China). Blood samples were obtained directly from the left ventricles of the mice and centrifuged.

For the biological analysis, subcutaneous adipose tissue was preserved in RNA Later solution (Invitrogen, Carlsbad, CA, USA) or liquid nitrogen (for a subsequent analysis of inflammatory response‐related and apoptosis‐associated genes or proteins). We also collected the serum of non‐stress and stressed mice for in vitro analyses [33].

### 2.6 Quantitative Polymerase Chain Reaction (qPCR)

For a quantitative real‐time polymerase chain reaction (qPCR) evaluation, whole RNA was harvested from SWAT with TRIZOL reagent (Invitrogen) according to the manufacturer's protocol. Subsequently, cDNAs were synthesized using a Reverse Transcriptase Kit (Zomanbio, Beijing, China) and subjected to a qPCR with the use of Power SYBR Green PCR Master Mix (Qiagen, Hilden, Germany) [34]. The gene expression levels were normalized to the housekeeping gene GAPDH, and we used an ABI 7300 Real‐Time PCR System (Applied Biosystems, Foster City, CA) to analyze the data. Table 1 provides the primer sequences for the target genes: monocyte chemotactic protein 1 (MCP‐1), interleukin‐6 (IL‐6), tumor necrosis factor‐alpha (TNF‐α), gp91^phox^, cytochrome *c*, caspase‐3, caspase‐9, Bcl‐2, Bax, stromal cell‐derived factor‐1 (SDF‐1), CXC chemokine receptor 4 (CXCR4), GLP‐1R, and glyceraldehyde 3‐phosphate dehydrogenase (GAPDH). All experiments were performed in triplicate.

**TABLE 1:** Mouse primer sequences used for the quantitative real‐time PCR.

Gene	Forward primer	Reverse primer
Bax	AGACAGGGGCCTTTTTGCTAC	AATTCGCCGGAGACACTCG
Bcl2	GCTACCGTCGTGACTTCGC	CCCCACCGAACTCAAAGAAGG
Caspase 3	GGGACTGATGAGGAGATGGC	GGGACTGGATGAACCACGAC
Caspase 9	TGCGGTGGTGAGCAGAAAGA	CTGGGAAGGTGGAGTAGGACA
CXCR4	CTTCTGGGCAGTTGATGCCAT	CTGTTGGTGGCGTGGACAAT
cyt *c*	TGCGGTGGTGAGCAGAAAGA	CTGGGAAGGTGGAGTAGGACA
IL‐6	CCAGTTTGGTAGCATCCATCATTTC	CCACTTCACAAGTCGGAGGCTTA
GLP‐1R	GATGCTGCCCTCAAGTGGAT	TAACGAACAGCAGCGGAACT
gp91^phox^	ACTTTCCATAAGATGGTAGCTTGG	GCATTCACACACCACTCAACG
MCP‐1	GGAGCCTAAGTTTGAGTTTGCTGTG	AGCAGCAGGTGTCCCAAAGA
SDF‐1	TGAGCGAGTACAACAAGGGC	GGCTGGTCATGGAAAGGACAG
TNF‐α	AGGCTGCCCCGACTACGT	GACTTTCTCCTGGTATGAGATAGCAAA
GAPDH	ATGTGTCCGTCGTGGATCTGA	ATGCCTGCTTCACCACCTTCT

Abbreviations: BAX, Bcl‐2 associated X protein; Bcl2, B‐cell lymphoma 2; CXCR4, CXC chemokine receptor 4; cyt *c*, cytochrome *c*; GAPDH, glyceraldehyde 3‐phosphate dehydrogenase; GLP‐1R, glucagon‐like peptide receptor; IL‐6, interleukin‐6; MCP‐1, monocyte chemotactic protein 1; SDF‐1, stromal cell‐derived factor 1; TNF‐α, tumor necrosis factor‐alpha.

### 2.7 Western Blot Analysis

Total proteins of SWAT tissue and cell lysates were isolated by RIPA supplemented with a protease and phosphatase inhibitor cocktail [35]. We used the Pierce BCA Protein Assay Kit (Solarbio) on a microplate reader to measure the protein concentrations. Equal amounts of proteins were separated by sodium dodecyl sulfate‐polyacrylamide gel electrophoresis (SDS‐PAGE), transferred to PVDF membranes, and blocked in 5% skim milk for 1 h. Primary antibodies against caspase‐8 (#4790), anti‐phosphorylated‐Akt‐(Ser473) (p‐Akts473, #4060S) and anti‐protein kinase B (AKT, #9272S), each from Cell Signaling Technology [CST], (Beverly, MA), anti‐phosphoinositide 3‐kinase (PI3K, #AF6242) and anti‐phospho‐PI3K p85 (Tyr458)/p55 (p‐PI3K, #AF3242, Affinity Biosciences, Cincinnati, OH) and β‐tubulin (#ab15246, Abcam, Cambridge, MA) (dilution 1:1000) were applied to the membranes and incubated overnight at 4°C according to the manufacturer's recommendations. After being washed with Tris‐buffered saline with 0.1% Tween 20 detergent (TBST), the membranes were treated with horseradish peroxidase (HRP)‐conjugated secondary antibodies (dilution 1:5000) for 2 h. Protein bands were visualized using the Amersham ECL Prime Western Blotting Detection Kit (GE Healthcare, Freiburg, Germany). Target protein levels quantified by western blots were normalized to the loading β‐tubulin levels or, in the case of phosphorylated proteins, to their unphosphorylated counterparts.

### 2.8 Histological and Immunofluorescence Staining

On stressed Day 14, SWAT from the seven groups of mice was fixed in 4% paraformaldehyde solution at 4°C for 24 h, followed by paraffin embedding and sectioning (4 μm thickness). For the histological analysis, tissue sections were stained with hematoxylin and eosin (H&E) standard staining. The size and distribution of adipocytes were quantified under high‐power magnification (400×) by three independent observers in a blinded manner. For the immunohistochemical analysis, tissue sections were incubated with primary antibodies against F4/80 (#71299S, CST) and iNOS (#ab15323, Abcam) for the assessment of specific histological markers. After three washes with phosphate‐buffered saline (PBS) containing 0.1% Tween‐20 (PBST), sections were incubated with appropriate secondary antibodies for 2 h at room temperature. Fresh tissue samples were used for dihydroethidium (DHE) staining (Beyotime, Shanghai, China) to assess the levels of reactive oxygen species (ROS) and for the detection of apoptosis using the One Step TUNEL Apoptosis Assay Kit (Beyotime), following the manufacturer's instructions. F4/80‐positive cells were counted in six independent sections from different mice, and the results are expressed as the number of F4/80^+^ cells per high‐power field [36].

### 2.9 Electron Microscopy Analysis of SWAT Mitochondria

For an electron microscopy analysis of SWAT mitochondria, SWAT was immediately fixed in a buffer containing 2.5% glutaraldehyde and 2% paraformaldehyde in 0.15 M sodium cacodylate buffer (pH 7.4). Tissues were further cut into approx. 1 mm^3^ cubes and immersed in fixative buffer overnight at 4°C. After washing, tissues were stained with 2% uranyl acetate for 1–2 h at 4°C, followed by dehydration in a graded series of ethanol solutions and then drying in acetone for 15 min at room temperature. The following day, adipose tissues embedded in Durcupan water‐soluble epoxy resin were placed in a 60°C oven for 36–48 h. Ultrathin (60 nm) sections were cut, stained with uranyl acetate and lead citrate, and imaged using a transmission electron microscope (model JEM‐1400, JEOL, Tokyo). The mitochondrial number and size were quantified at 5000× magnification by counting pixels with the use of Image J software. For each mouse, six mitochondrial cross‐sectional areas from six tissue sections were analyzed.

### 2.10 Flow Cytometry Analysis

On stressed Day 14, bone marrow was incubated with antibodies such as CD45^+^, F4/80,^+^ and CD11b^+^ at constant temperature for 1 h; then, 2 mL of FACS lysis buffer was added, and room temperature was 10 min. Then, the PBS buffer was added to the suspension, and the analysis was carried out by flow cytometry.

### 2.11 Co‐Culture of 3T3‐L1 and RAW 264.7

Co‐cultures of 3T3‐L1 cells and RAW 264.7 cells were performed using Transwell chambers. 3T3‐L1 cells were seeded in the lower chamber, and RAW 264.7 cells were placed in the upper chamber. The cells were stimulated with interferon‐gamma (IFN‐γ) (100 ng/mL, Peprotech, Rocky Hill, NJ) and lipopolysaccharide (LPS) (10 ng/mL, Sigma, St. Louis, MO). 5% NS‐serum/DMEM (Dulbecco's modified Eagle's medium) was added to the upper chamber, and 5% NS‐serum/DMEM or 5% S‐serum/DMEM was added to the lower chamber. After incubation, the bottom 3T3‐L1 cells were collected for further analyses, including western blotting and immunofluorescence. Following fixation and the removal of inner chamber cells, the out‐chamber cells of each well were stained with crystal violet for the assessment of cell migration ability.

### 2.12 Cell Culture

3T3‐L1 preadipocytes were purchased from Zhong Qiao Xin Zhou Biotechnology (Shanghai, China). The 3T3‐L1 cells were cultured with 10% fetal bovine serum (FBS) in DMEM (Viva Cell, Shanghai, China) at 37°C in a humidified atmosphere of 5% CO_2_ and 95% air. The 3T3‐L1 cells were exposed to one of the following conditions for 24 h in the lower chamber of a Transwell system: 5% NS‐serum (from non‐stressed mice) or 5% S‐serum (from stressed mice). RAW 264.7 cells were seeded in the upper chamber and stimulated with or without LPS (10 ng/mL) and IFN‐γ (100 ng/mL).

In a separate experiment, cells were cultured with or without 30 nM exenatide or the PI3K/AKT inhibitor LY294002 (20 μM, APExBIO, Technology, Houston, TX) in the presence of 5% S‐serum in DMEM for 24 h. Cellular assays for targeted protein expression were then performed. All cell culture assays were performed and repeated at least three times.

### 2.13 Isolation of Primary Adipocytes

For the isolation of primary adipocytes, WT and DPP4 knockout mice were exposed to either non‐stress or chronic stress conditions for 2 weeks. SWAT was harvested from each group, minced into small fragments (~1 mm^3^), and digested in DMEM supplemented with 1.5 mg/mL collagenase type I (Invitrogen) at 37°C for 90 min. The digested mixture was filtered with a 200‐μm nylon mesh, and cell sediments (preadipocytes) were collected by 1500 rpm centrifugation for three consecutive 10‐min intervals. The same groups of preadipocytes were then cultured with serum from the same groups.

### 2.14 DCFH‐DA, MitoTracker, JC‐1 and Mito SOX Staining

For the staining of cells by JC‐1 and mitochondrial superoxide (Mito SOX), adipocytes were co‐cultured as described above, washed three times with PBS, and then stained with 2′,7′‐dichlorodihydrofluorescein diacetate (DCFH‐DA), JC‐1, Mito‐SOX, or MitoTracker (Beyotime) according to the manufacturer's protocols.

### 2.15 Statistical Analyses

All results are presented as the mean ± SEM (standard error of the mean). Two‐tailed unpaired Student's *t*‐test (for comparisons of two groups) and a one‐way analysis of variance (ANOVA) (for comparisons of ≥ 3 groups) followed by Tukey's post hoc test were used for the statistical analyses. The body weight (BW) data were subjected to a two‐way repeated‐measures ANOVA and Bonferroni's post hoc test. After the data's distribution status was identified, the data were applied to the statistical analyses. The mice had been randomly allocated to the experimental groups. All of the morphometric evaluations were done by two observers in a blind manner, and the values the observers obtained were averaged. The statistical analyses were performed using GraphPad Prism ver. 9.0.0 software (GraphPad, La Jolla, CA). Probability (*p*)‐values < 0.05 were considered significant.

## 3 Results

### 3.1 The Network Pharmacology Analysis for Target Molecule Identification

We applied a network pharmacology approach to explore the potential molecular targets of exenatide for inflammation and dysfunction in adipose tissue of mice subjected to chronic stress. A total of 171 exenatide‐related genes in the SwissTargetPrediction database were identified, and 5470 genes linked to chronic stress‐related adipose inflammation were obtained from the GeneCards database. A Venn diagram generated by the bioinformatics platform revealed 138 overlapping targets (Figure 1A). As shown in Figure 1B, the results of the gene ontology (GO) pathway enrichment analysis suggested processes associated with the inflammatory response.

To investigate the effects of exenatide on adipose inflammation and chronic stress, we imported the 138 shared molecule targets into the STRING database and constructed a protein–protein interaction (PPI) network (Figure 1C) using Cytoscape ver. 3.10.2. The node sizes and colors were adjusted according to degree values, with darker colors indicating higher degrees. Caspase‐3, PI3KR1, and PIK3CD were identified as high‐degree nodes. We conducted a KEGG pathway analysis to identify the signaling pathways implicated by the anti‐inflammatory molecule targets of exenatide, uncovering 138 statistically significant pathways. A further KEGG enrichment analysis using the DAVID database pinpointed 15 relevant pathways (Figure 1D), with cancer‐related, apoptosis, and chemokine signaling pathways ranking highest.

The Compounds‐Key‐Targets‐Pathway Network also indicated exenatide's involvement in apoptosis pathways and its association with targets such as GLP‐1R, CXCR4, Bcl2, caspase 3, and caspase 8 (Figure 1E). Overall, our observations indicated that exenatide might alleviate adipose inflammation and dysfunction through the PI3K/AKT and apoptosis signaling pathways, providing a theoretical basis for future mechanistic explorations and therapeutic applications.

### 3.2 Chronic Stress Inactivated the PI3K/AKT Signaling Pathway and Increased ROS Levels in SWAT

**FIGURE 2:**
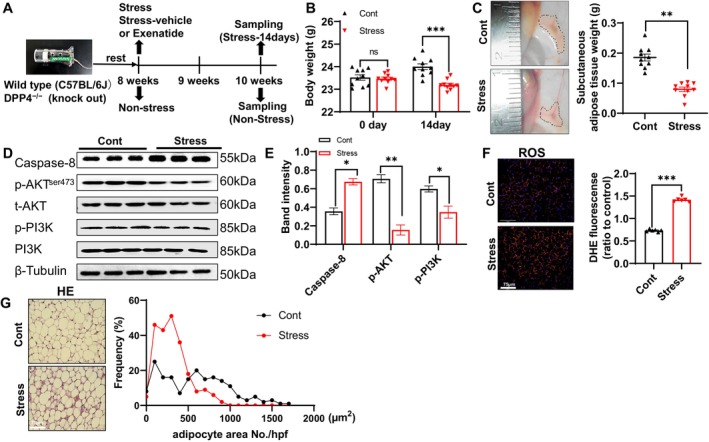
The effects of the 2‐week chronic stress on the production of reactive oxygen species (ROS) and adipose remodeling. (A) Timeline of the 2‐week chronic stress protocol and subcutaneous white adipose tissue (SWAT) sampling. (B, C) Photographs of SWAT and quantitative data showing the weight data of the body weight (BW) and SWAT in the two experimental groups (*n* = 10/group). (D, E) Representative immunoblot images and combined quantitative data showing the protein levels of caspase‐8, p‐AKT, and p‐PI3K in both groups (*n* = 3/group). (F) ROS staining images and the combined quantitative data show the ROS production (*n* = 6/group). (G) H&E staining images and the combined quantitative data depicting the frequency of adipocyte area in the two experimental groups (*n* = 6/group). Scale bar: 75 μm. Data are mean ± SEM. NS: Not significant. Statistical significance was assessed by two‐way ANOVA (B) or unpaired Student's *t*‐test (C, E, F, right panels). *p < 0.05, **p < 0.01, ***p < 0.001.

The restraint stress protocol used in this study has been confirmed as being useful to evaluate stress‐related inflammatory and thrombotic cardiovascular disorders [1, 33]. Eight‐week‐old DPP4^+/+^ mice were subjected to chronic stress for 14 days in the present experiment (Figure 2A); on Day 14 of the stress period, the body weights (BWs) and SWAT mass values of the stressed mice were markedly lower than those of the non‐stressed mice (Figure 2B,C).

**FIGURE 3:**
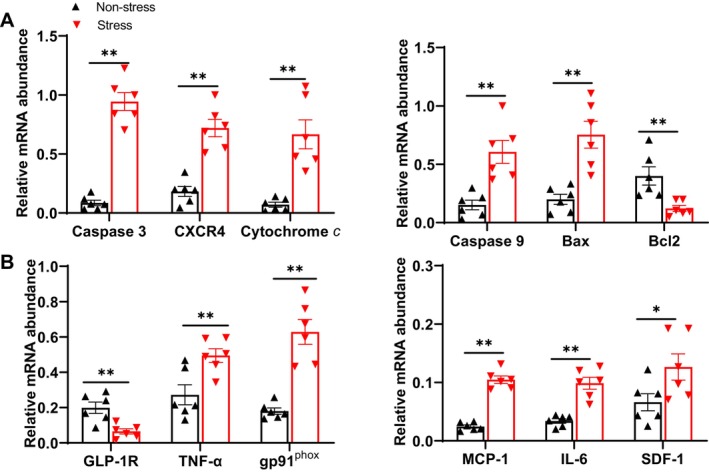
The effects of 2‐week stress on the investigated gene expressions in the SWAT. (A,B) The results of the quantitative real‐time PCR showing the levels of apoptosis‐related (caspase 3, caspase 9, Bcl2, Bax, cytochrome *c*), oxidative stress‐related (gp91^phox^), and inflammation‐related (SDF‐1, CXCR4, TNF‐α, MCP‐1, GLP‐1R, and IL‐6) genes in the SWAT of both groups (*n* = 6/group). Data are mean ± SEM. *p < 0.05, **p < 0.01 by unpaired Student's *t*‐test.

To further identify the molecular changes in adipose remodeling in response to stress, we analyzed the expression levels of inflammation‐, oxidative stress, and apoptosis‐related molecules. The qPCR results demonstrated significantly increased levels of molecules related to inflammation (SDF‐1, CXCR4, TNF‐α, IL‐6 and MCP‐1), oxidative stress (gp91^phox^) and apoptosis (cytochrome *c*, caspase‐3, casepase‐9, and Bax) in the SWAT of the stressed mice compared to that of the non‐stressed mice (Figure 3A,B). Bcl2 and GLP‐1R genes were also significantly downregulated in the stressed SWAT (Figure 3A,B). The western blotting data revealed that the level of an apoptosis‐related protein (caspase‐8) was significantly increased by chronic stress, whereas the protein levels of p‐AKT and p‐PI3K were reduced in the stressed SWAT (Figure 2D,E). The ROS staining data demonstrated that chronic stress increased the level of oxidative stress (Figure 2F), and the quantitative data from the H&E staining revealed a marked shift to the lower frequency of large adipocytes (> 600 μm^2^) in the SWAT from stressed mice (Figure 2G).

### 3.3 DPP4 Deletion Mitigated Inflammation and Apoptosis in the Stressed SWAT

**FIGURE 4:**
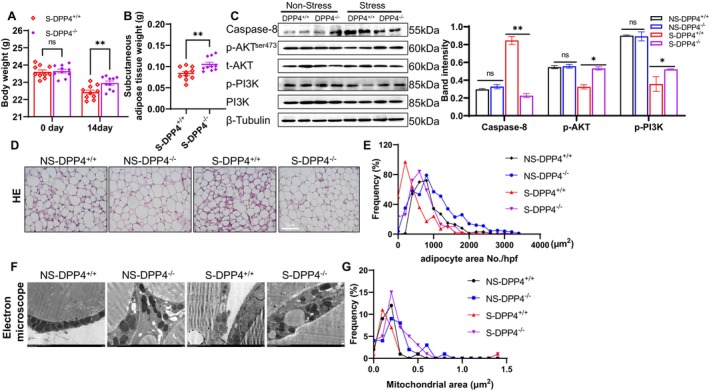
DPP4 deletion prevented the inactivation of PI3K‐AKT signaling in the stressed SWAT. DPP4^+/+^ and DPP4^−/−^ mice were randomly assigned to non‐stress groups (NS‐DPP4^+/+^ and NS‐DPP4^−/−^) and stress groups (S‐DPP4^+/+^ and S‐DPP4^−/−^) and subjected to the SWAT sampling at day 14 after stress. (A, B) Quantitative data of the weights of BW and SWAT in the two experimental groups (*n* = 10/group). (C) Representative immunoblot images and combined quantitative data showing the protein levels of caspase 8, p‐AKT, and p‐PI3K in four groups (*n* = 4/group). (D, E) Representative H&E staining images and the combined quantitative data showing the frequencies of adipocyte sizes (μm^2^) in the four groups (*n* = 6/group). Scale bar: 75 μm. (F, G) Representative mitochondrial transmission electron microscope images and the combined quantitative data showing the frequency of mitochondrial areas in the four groups (*n* = 3/group). Scale bar: 0.2 μm. Results are mean ± SEM. Statistical significance was assessed by two‐way ANOVA (A), unpaired Student's *t*‐test (B), or one‐way ANOVA (C, G). *p < 0.05, **p < 0.01.

As shown in Figure 4A, compared to the values of the DPP4^+/+^‐stressed mice, the body weight and SWAT weight were higher in the DPP4^−/−^‐stressed mice. Our group's earlier investigations demonstrated that elevated plasma DPP4 and TNF‐α levels and reduced GLP‐1 levels in stressed mice were reversed by the genetic and pharmacological inhibitions of DPP4 [18, 21]. Our present study's western blot results revealed that DPP4 deletion produced a beneficial effect on the harmful changes in the levels of p‐AKT, p‐PI3K, and caspase‐8 in the stressed SWAT samples (Figure 4C). The quantitative analysis of H&E staining revealed that DPP4 knockout increased the frequency of large adipocytes (> 800 μm^2^) and decreased the number of CLSs in the stressed mice (Figure 4D,E).

**FIGURE 5:**
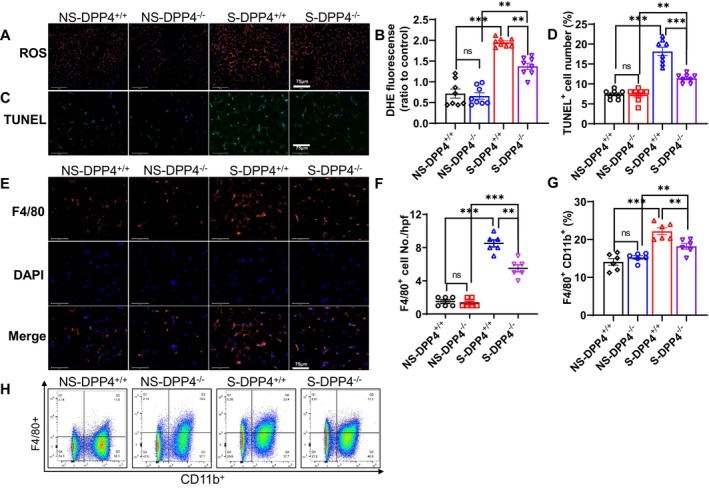
DPP4 deletion lowered the production of ROS, apoptosis, and macrophage infiltration in the stressed SWAT. (A, B) Representative fluorescence images and combined quantitative data showing the ROS production in four groups (*n* = 7/group). (C, D) Representative fluorescence images and combined quantitative data showing the number of TUNEL^+^ cells in four groups (*n* = 8/group). (E–H) Representative fluorescence images and quantitative data showing the numbers of F4/80^+^ cells in adipose tissue and bone marrow. Scale bar: 75 μm. Results are mean ± SEM. Statistical significance was assessed by one‐way ANOVA (B, D, F, G). *p < 0.05, **p < 0.01, ***p < 0.001.

**FIGURE 6:**
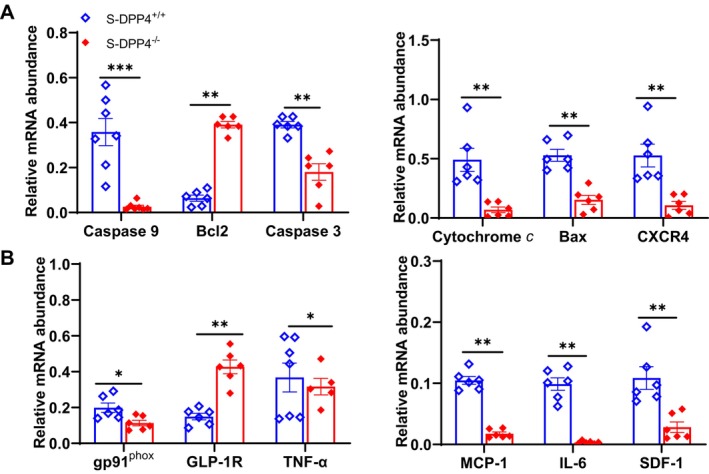
DPP4 deficiency ameliorated the investigated gene expressions in the mice that underwent the 2‐week stress protocol. (A, B) Quantitative real‐time PCR showing the expressions of apoptosis‐related (caspase 3, caspase 9, Bcl2, Bax, cytochrome *c*), oxidative stress‐related (gp91^phox^), and inflammation‐related (SDF‐1, CXCR4, TNF‐α, MCP‐1, GLP‐1R, and IL‐6) genes in the stressed SWAT of both groups (*n* = 6/group). Data are mean ± SEM. *p < 0.05, **p < 0.01 by unpaired Student's *t*‐test.

The transmission electron microscopy analysis of SWAT mitochondria revealed that the frequency distribution of mitochondrial areas was significantly larger (> 0.25μm^2^) in the stressed DPP4^−/−^ SWAT compared to that of the stressed DPP4^+/+^ mice (Figure 4F,G). The ROS and TUNEL staining results demonstrated that subjecting mice to the chronic stress protocols enhanced the oxidative stress production and apoptosis (Figure 5A–D) and increased the number of F4/80^+^ macrophages in adipose tissue and bone marrow (Figure 5E–H). These effects were still reversed by DPP4 deficiency. The qPCR data revealed that DPP4 deficiency markedly lowered the expressions of IL‐6, TNF‐α, MCP‐1, gp91^phox^, cytochrome *c*, caspase 3, caspase 9, and Bax but restored the expressions of GLP‐1R and Bcl2 (Figure 6A,B). However, there was no obvious difference in those parameters between the non‐stressed DPP4^+/+^ and DPP4^−/−^ mice.

### 3.4 The GLP‐1 Receptor Agonist Exenatide Exerted an Adipose Benefit in DPP4 ^+/+^ Mice Under Stress Conditions

**FIGURE 7:**
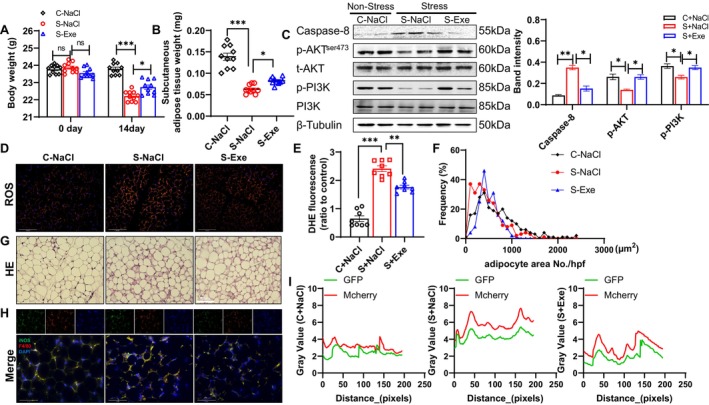
The pharmacological GLP‐1 receptor agonist exenatide prevented adipose wasting and PI3K/AKT signaling inactivation in WT mice that were subjected to 2 weeks of stress. (A, B) Body weights and SWAT weights in the three experimental groups (*n* = 10/group). (C) The protein levels of caspase 8, p‐AKT, and p‐PI3K in the three experimental groups (*n* = 4/group). (D, E) Representative fluorescence images and combined quantitative data of the production of ROS in the three groups. (F, G) H&E staining images and the combined quantitative data showing the frequencies of adipocyte areas in the three groups. (H, I) Fluorescence images and quantitative data showing the colocalization of F4/80^+^ macrophages and iNOS. Scale bar: 75 μm. Results are mean ± SEM. Statistical significance was assessed by two‐way ANOVA for (A), and one‐way ANOVA for (B, C, E). *p < 0.05, **p < 0.01, ***p < 0.001.

The BW and SWAT mass data of the S‐Exe group were significantly higher than those of the S‐NaCl group (Figure 7A,B). The GLP‐1 receptor agonist exenatide rectified the alterations in the levels of p‐AKT, p‐PI3K, and caspase‐8 in the stressed SWAT (Figure 7C). The representative H&E staining images and quantitative data in panels F and G of Figure 7 demonstrate that the exenatide treatment markedly increased the frequency of large (> 500 μm^2^) adipocyte areas in the SWAT. Consistently, the results of the quantitative data and immunofluorescence staining showed a reduction in the ROS production (Figure 7D,E) and the colocalization intensities of F4/80 and inducible nitric oxide synthase (iNOS) (Figure 7H,I) in the SWAT of the S‐Exe group.

**FIGURE 8:**
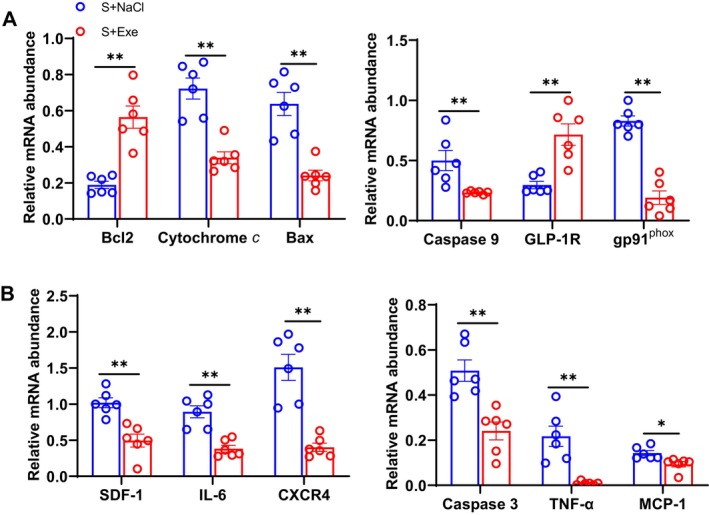
Exenatide mitigated the targeted gene expressions in stressed SWAT. (A, B) Quantitative real‐time PCR showing the expressions of apoptosis‐related (caspase 3, caspase 9, Bcl2, Bax, cytochrome *c*), oxidative stress‐related (gp91^phox^), and inflammation‐related (SDF‐1, CXCR4, TNF‐α, MCP‐1, GLP‐1R, and IL‐6) genes in the stressed SWAT of both groups (*n* = 6/group). Data are mean ± SEM. *p < 0.05, **p < 0.01 by unpaired Student's *t*‐test.

**FIGURE 9:**
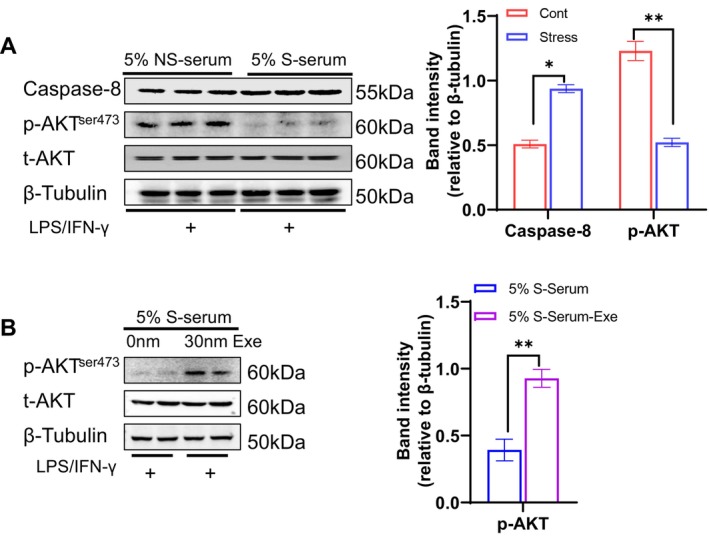
Stress serum increased the level of caspase‐8 protein and reduced the level of p‐AKT^ser473^ protein. (A) Following co‐culture as described for Figure 10, the lysates isolated from the 3T3‐L1 cells of the well bottoms were subjected to a western blotting assay. Representative immunoblot images and combined quantitative data show the levels of caspase‐8 and p‐AKT protein in both groups (*n* = 3/group). (B) Following co‐culture as described above, 3T3‐L1 cells were treated with 30 nM exenatide at the indicated time points and subjected to a western blotting assay. Representative images and combined quantitative data show the levels of p‐AKT protein in the two experimental groups (*n* = 4/group). Data are mean ± SEM. Statistical significance was assessed by unpaired Student's *t*‐test (A, B). *p < 0.05, **p < 0.01.

Similar to DPP4 deletion, exenatide lowered the levels of cytochrome c, Bax, caspase‐8/−9, TNF‐α, MCP‐1, SDF‐1, IL‐6, and gp91^phox^ mRNA but enhanced the levels of Bcl2 and GLP‐1R mRNA in the stressed SWAT (Figure 8A,B). As shown in Figure 9B, the results of an in vitro experiment revealed that exenatide exerted a beneficial effect on p‐AKT protein alterations in 3T3‐L1 cells under 5% S‐serum conditions. These findings indicate that a GLP‐1/GLP‐1R axis functions as an important positive mediator of stress‐related adipose inflammatory actions and apoptosis in vivo and in vitro.

### 3.5 S‐Serum Inactivated PI3K‐AKT Signaling in 3T3‐L1 Cells

**FIGURE 10:**
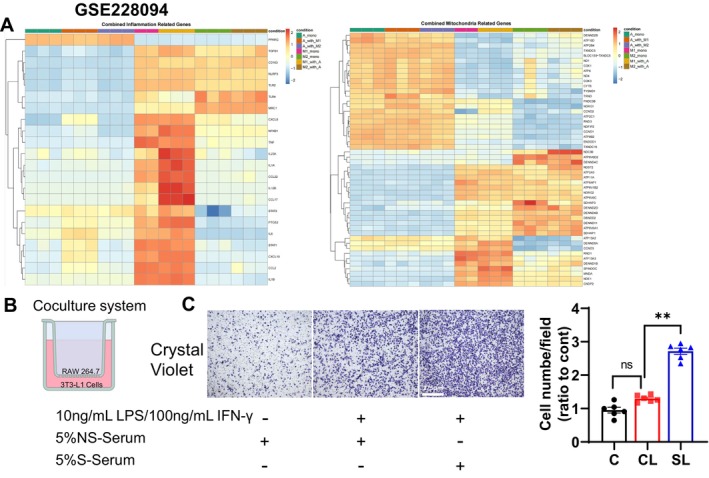
The interaction of adipocytes and macrophages to stimulate inflammation in the co‐culture model. (A) Data mining of GSE228094. (B) Schematic of the co‐culture mode: RAW264.7 cells were seeded in the upper chambers and 3T3‐L1 cells were seeded in the lower chambers. (C) The crystal violet images and the quantitative number of cells showing the migrated RAW264.7 cells of the out‐membranes induced by 5% NS‐serum/DMEM and 5% S‐serum/DMEM in the presence or absence of LPS/IFN‐γ (*n* = 6/group). Data are mean ± SEM. Statistical significance was assessed by one‐way ANOVA (C). NS: Not significant. **p < 0.01.

We downloaded RNA sequencing data from the GEO database, acquiring six datasets (GSE228094): adipocyte mono (A_mono), adipocyte with M1 macrophages (A_with_M1), adipocyte with M2 macrophages (A_with_M2), M1 mono, M2 mono, M1 with adipocytes (M1_with_A) and M2 with adipocytes (M2_with_A) [37]. The mitochondria‐ and inflammation‐associated gene enrichment heatmap showed no significant differences among the following three groups: A_mono, A_with_M1, and A_with_M2. However, the expression levels were significantly higher in the M1_with_A group and M2_with_A group compared to the A_mono group, with the M1_with_A group exhibiting the most pronounced upregulation (Figure 10A), indicating that an interaction between adipocytes and inflammatory macrophages may be an important step to promote adipose inflammation.

**FIGURE 11:**
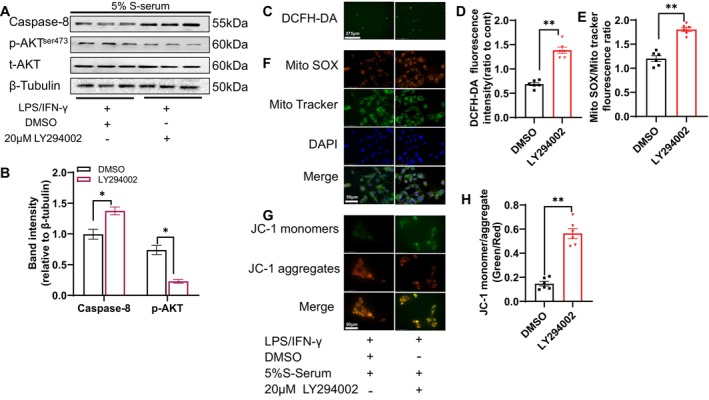
The PI3K/AKT inhibitor LY294002 exacerbated the alterations in the signal proteins and oxidative stress production. Following co‐culture for 24 h as described for Figure 10, the 3T3‐L1 cells were treated with LPS/IFN‐γ and/or LY294002 at the indicated concentrations and were then subjected to western blotting and immunofluorescence assays. (A, B) The effects of stress‐serum and LY294002 on caspase‐8 and p‐AKT proteins in 3T3‐L1 cells as revealed by protein blotting. (C, D) Representative images and combined quantitative data show the intracellular ROS production (scale bar: 275 μm, *n* = 6/group). (E, F) Representative images and combined quantitative data show the mitochondrial oxidative stress‐related Mito SOX staining intensities (scale bar: 50 μm, *n* = 6/group). (G, H) Representative images and combined quantitative data show the apoptotic JC‐1 staining intensities in the four groups (scale bar: 50 μm, *n* = 6/group). Data are mean ± SEM. Statistical significance was assessed by unpaired Student's *t*‐test (B, D, E, H). *p < 0.05, **p < 0.01.

To explore both of these interactions in vitro, we used a co‐culture system of 3T3‐L1 and RAW264.7 cells as outlined in the Materials and Methods section (Figure 10B). Compared to the 5% NS‐serum group, the quantitative data of the out‐well chamber cells assessed by crystal violet staining revealed that the 5% S‐serum significantly enhanced the macrophage (RAW264.7) migration ability in the presence of LPS/IFN‐γ (Figure 10C). We next subjected the lysates isolated from the bottom 3T3‐L1 cells to western blotting assays, and as anticipated, the 5% S‐serum exerted a harmful effect on caspase‐8 and AKT in 3T3‐L1 cells (Figure 9A). In addition, PI3K‐AKT signaling blockade by a PI3K‐AKT inhibitor (LY294002) exacerbated the stress‐mediated changes in the target proteins (Figure 11A,B). In a similar manner, LY294002 increased S‐serum‐induced intracellular ROS production as well as the production of Mito SOX and mitochondrial apoptosis as revealed by JC‐1 staining (Figure 11C–H). Collectively, these results indicate that an interaction of adipocytes and macrophages may facilitate inflammation and apoptosis through the regulation of PI3K‐AKT signaling in adipocytes under S‐serum conditions.

### 3.6 DPP4 Deletion Modified the S‐Serum‐Induced Adipocyte Apoptosis in SWAT

**FIGURE 12:**
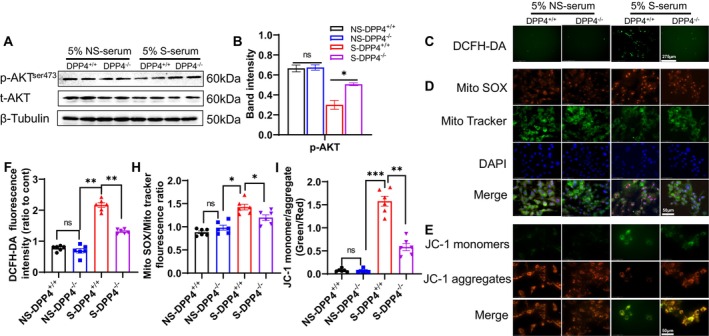
DPP4 deletion reduced the preadipocyte oxidative stress production and apoptosis in response to S‐serum. DPP4^+/+^ and DPP4^−/−^ mice were divided into non‐stress (NS‐DPP4^+/+^ and NS‐DPP4^−/−^) and stress groups (S‐DPP4^+/+^ and S‐DPP4^−/−^) and subjected to the isolation of preadipocytes from the SWAT at day 14 after the stress protocol. The preadipocytes were then cultured in 5% NS‐serum or 5% S‐serum for 24 h and subjected to western blotting and immunofluorescence analyses (DCFH‐DA, Mito SOX/MitoTracker, and JC‐1 monomers/aggregates). (A, B) Representative immunoblot images and combined quantitative data showing the protein levels of p‐AKT in the four groups (*n* = 4/group). Representative images (C, D) and combined quantitative data (F, H) show the intracellular ROS production (scale bar: 275 μm) and mitochondrial oxidative stress represented by Mito SOX (scale bar: 50 μm, *n* = 6/group). (E, I) Representative images and combined quantitative data of the JC‐1 staining in the four groups (scale bar: 50 μm, *n* = 6/group). Data are mean ± SEM. Statistical significance was assessed by one‐way ANOVA (B, F–I). NS: Not significant. *p < 0.05, **p < 0.01, ***p < 0.001.

DPP4^+/+^ and DPP4^−/−^ mice were subjected to either non‐stress or chronic stress conditions for 2 weeks. Preadipocytes were then harvested from their SWAT and cultured in the presence of 5% NS‐serum and 5% S‐serum. Consistent with in *vivo* results, DPP4^−/−^ increased the levels of p‐AKT protein in SWAT‐derived preadipocytes under the S‐serum condition (Figure 12A,B). In a similar manner, S‐serum markedly increased the intracellular and mitochondrial ROS production as well as mitochondrial apoptosis, and these effects were diminished by DPP4 deletion (Figure 12C–I), indicating that DPP4^−/−^ SWAT‐derived preadipocytes were resistant to S‐serum.

## 4 Discussion

This study focused on the critical role(s) of the DPP4‐GLP‐1/GLP‐1R‐mediated interaction between the adipocytes and macrophages in adipose inflammation and dysfunction in mice under stress conditions. The main significant finding of this study is that mice with DPP4 deletion were resistant to chronic stress‐related metabolic adipose disorder. At the cellular and molecular levels, the 2‐week stress caused harmful changes in the SWAT, and these effects were reversed by DPP4 deletion and GLP‐1R activation in the following three ways: (1) reducing the body weights, SWAT mass, and adipocyte size; (2) inactivating the PI3K/AKT signaling pathway, accompanied by reductions of GLP‐1R and Bcl2 proteins and enhancements of genes and/or proteins related to inflammation (TNF‐α, MCP‐1, SDF‐1, and IL‐6), apoptosis (Bax, caspase 8, caspase 9, and cytochrome *c*), and oxidative stress (gp91^phox^) in the stressed SWAT; and (3) increased macrophage infiltration and mitochondrial morphological changes as well as increased intracellular and mitochondrial oxidative stress production in the stressed SWAT. These alterations were markedly rectified by DPP4 deletion as well as the exenatide‐mediated pharmacological GLP‐1R activation. In vitro, the data of the co‐culture model using 5% S‐serum containing exenatide, LPS/IFN‐γ, and LY294002 at the indicated concentrations confirmed that an interaction of adipocytes and macrophages resulted in the inactivation of PI3K/AKT signaling, accompanied by increased intracellular and mitochondrial oxidative stress production and apoptosis, providing evidence and a mechanistic explanation for the involvement of GLP‐1/GLP‐1R‐mediated PI3K/AKT signaling in adipocyte and macrophage cross‐talk toward the development of metabolic adipose disorder.

DPP4 inhibition enhances GLP‐1 signaling, resulting in pleiotropic effects [22]. Similar to findings in DPP4‐deficient mice, our present results demonstrate that exenatide treatment effectively ameliorates adipose inflammation‐related molecules and macrophage infiltration in stressed adipose tissue. A previous review highlighted the role of the GLP‐1/GLP‐1R agonists in the reduction of inflammation and apoptosis and the improvement of cardiovascular function [38]. Moreover, preclinical and clinical studies have shown that brown adipose tissue (BAT) is a new potential target for GLP‐1R agonists in the treatment of obesity [39]. Together, these past and present data suggest that an upregulation of adipose GLP‐1/GLP‐1R signaling by DPP4 inhibition may be a common mechanism for treating adipose inflammation and dysfunction under CPS conditions.

Chronic psychological stress has been demonstrated to amplify inflammatory responses across various tissues, including adipose and cardiovascular tissues [1, 17]. Our present quantitative qPCR and histological data revealed that CPS led to SWAT wasting, adipose inflammation, and apoptosis. These effects were associated with adverse changes in the expressions of pro‐inflammatory chemokines (SDF‐1, CXCR4, IL‐6, TNF‐α and MCP‐1) and macrophage infiltration (F4/80). Notably, these changes were significantly attenuated by DPP4 knockout in stressed mice. Consistent with these findings, it has been shown that stress leads to inflammation and metabolic disorders and that IL‐6 is a key signal for stress induction [40]. Obesity was reported to stimulate the synthesis of hepatocytes and the secretion of DPP4, which act in conjunction with plasma factor Xa to promote inflammatory adipose tissue macrophages and insulin resistance [7]. Our present experiments revealed that the levels of p‐PI3K and p‐AKT were significantly decreased in the stressed mice, whereas DPP4 deficiency rectified their expressions. Notably, we observed that the pharmacological GLP‐1 agonist exenatide mimicked the beneficial effects mediated by DPP4 knockdown in vivo and/or in vitro. Moreover, the PI3K/AKT inhibitor LY294002 was observed to further increase the ROS and Mito SOX levels of the stress effect. It has been shown that intercellular mitochondria can be transferred from neighboring adipocytes to macrophages, but this role is impaired in obesity [41]. In humans with obesity, macrophage activation contributes to systemic low‐grade inflammation via the PI3K/mTOR pathway [42]. In light of the beneficial effects of DPP4 inhibition on metabolic disorders, including obesity and diabetes mellitus [43], we propose that DPP4‐GLP‐1/GLP‐1R may be a pivotal regulator of stress‐induced adipose inflammation and dysfunction via the negative modulation of the PI3K/AKT signaling pathway.

Over the past two decades, our group has observed that under stress conditions, the circulating levels of DPP4 catalytic activity increase in the brain, subcutaneous adipose tissue, and other types of tissue [19]. Other researchers have noted that DPP4 inhibition lowered the expressions of factors involved in inflammation (IL‐1β) and oxidative stress (p47^phox^, p67^phox^) and increased the expression of an anti‐oxidative stress factor (SOD‐1/2) in stressed mice [20, 33]. Our present results demonstrate that chronic stress induced significant increases in murine gp91^phox^ and ROS levels, characterized by a marked upregulation of caspase‐8 factor, which in turn results in increased mitochondrial ROS production and apoptosis. Importantly, these effects can be reversed by the GLP‐1 agonist exenatide. Apoptosis is one of the forms of programmed cell death and one of the key pathogenic mechanisms of chronic stress‐related diseases [21, 33]. It was reported that caspase‐8 reduced pro‐survival Bcl‐2 transcription and increased the iNOS level, thus facilitating Bax and Bak signaling [44]. Our present experiments revealed that the expressions of apoptosis‐related genes (caspase 3, 8, and 9, cytochrome *c*, and Bax) increased significantly after stress, whereas the expression of Bcl2 decreased, and these changes were reversed by DPP4 deletion in the stressed SWAT. Collectively, these findings suggest that in mice under our experimental conditions, (i) DPP4 inhibition mediates the attenuation of oxidative stress, and (ii) apoptosis might contribute to the improvement of adipose metabolic changes.

SDF‐1 is constitutively expressed in various organs including adipose tissue, the heart, and bone marrow [45, 46]. CXCR4 is the principal specific receptor for SDF‐1. This signaling cascade regulates the expressions of cytokines and chemokines, and SDF‐1 has been shown to upregulate the secretion of TNF‐α mRNA and protein, which is directly correlated with subsequent apoptosis [47]. Another of our research group's studies shows that CTSS (cathepsin S) functions as a regulator of SDF‐1/CXCR4‐mediated inflammatory responses in mice exposed to chronic stress [48]. In the present study, chronic stress increased the levels of SDF‐1/CXCR4 genes in the SWAT of the mice, and these changes were reversed by DPP4 deficiency and by the GLP‐1 agonist exenatide, suggesting that DPP4/GLP‐1 may act as a regulator of SDF‐1/CXCR4‐mediated inflammatory responses in stressed mice.

Another implication of our research is the potential utility of elevated pro‐inflammatory factors (IL‐6, TNF‐α, and MCP‐1) as biomarkers for predicting stress in animals. The results of our present and prior studies suggest that DPP4 and GLP‐1 are sensitive to chronic stress and could serve as valuable indicators for assessing adipose tissue injury in animals exposed to such stress [16, 17, 18, 19].

However, it is important to acknowledge several limitations of the present study. For example, the chronic stress model that we used is an animal‐based model that has inherent limitations, as it cannot fully replicate the complexity of human psychological stress. Second, the mitochondrial transfer between adipocytes and macrophages in the adipose inflammation of animals under chronic stress remains to be investigated. Third, the adipose‐specific benefits conferred by DPP‐4 inhibition were not investigated in this study. In summary, our findings help explain how stress causes adipose metabolic alterations, and they also suggest roles of the DPP4/GLP‐1/GLP‐1R axes in the stress‐related adipose inflammation and dysfunction processes. In mice exposed to stress, the inactivation of the GLP‐1R‐PI3K/AKT signaling pathway caused by an elevated level of DPP4 facilitates an interaction between adipocytes and macrophages, leading to adipocyte intracellular and mitochondrial oxidative stress production and apoptosis linked to adipose inflammation and dysfunction. Genetic DPP4 deletion and GLP‐1R signaling activation rectified the imbalance between DPP4 and GLP‐1 in the adipose tissues of mice, emphasizing the notion that one or both of these approaches might be a potential avenue toward clinical treatment for metabolic adipose disorder in humans living with chronic psychological stress.

## Author Contributions

Meiping Zhang: conceptualization, formal analysis, investigation, methodology, writing – original draft. Huazhen Wang: investigation, data curation, methodology. Xiangdan Li: investigation and methodology (electric microscopies). Shangzhi Shu: data curation, methodology. Xueling Yue: data curation, methodology. Jinshun Piao: methodology (animal handling). Miao Li and Songzhen Zhao researched the morphological data and assisted with the chronic stress mouse models. Xianglan Jin: validation, writing – review and editing. Yongshan Nan: validation, writing – review and editing. Xian Wu Cheng conceived the project, designed and performed experiments, interpreted data, and wrote, revised, and critically reviewed the article. All authors read and approved the article.

## Ethics Statement

The animal study protocols were approved by the Institutional Animal Care and Use Committees of Yanbian University (protocol no. YD20240430002) for the experiments with DPP4^+/+^ and DPP4^−/−^ mice and performed in accordance with the Guide for the Care and Use of Laboratory Animals published by the U.S. National Institutes of Health.

## Conflicts of Interest

The authors declare no conflicts of interest.

## Data Availability

The original contributions presented herein are included in the article. Further inquiries can be directed to the corresponding authors.
